# WEClustering: word embeddings based text clustering technique for large datasets

**DOI:** 10.1007/s40747-021-00512-9

**Published:** 2021-09-07

**Authors:** Vivek Mehta, Seema Bawa, Jasmeet Singh

**Affiliations:** Computer Science and Engineering Department, Thapar Institute of Engineering and Technology, Patiala, Punjab 147001 India

**Keywords:** Document clustering, Text mining, BERT, Semantic clustering, Pattern recognition, Big data

## Abstract

A massive amount of textual data now exists in digital repositories in the form of research articles, news articles, reviews, Wikipedia articles, and books, etc. Text clustering is a fundamental data mining technique to perform categorization, topic extraction, and information retrieval. Textual datasets, especially which contain a large number of documents are sparse and have high dimensionality. Hence, traditional clustering techniques such as K-means, Agglomerative clustering, and DBSCAN cannot perform well. In this paper, a clustering technique especially suitable to large text datasets is proposed that overcome these limitations. The proposed technique is based on word embeddings derived from a recent deep learning model named “Bidirectional Encoders Representations using Transformers”. The proposed technique is named as WEClustering. The proposed technique deals with the problem of high dimensionality in an effective manner, hence, more accurate clusters are formed. The technique is validated on several datasets of varying sizes and its performance is compared with other widely used and state of the art clustering techniques. The experimental comparison shows that the proposed clustering technique gives a significant improvement over other techniques as measured by metrics such Purity and Adjusted Rand Index.

## Introduction

Nowadays, a huge amount of textual data exists in digital form. For example, millions of articles in a year are published in thousands of journals of English language alone [20] and their numbers are continuously increasing. For example, there are more than 37,000 articles on just the COVID-19 topic in Elsevier’s repository alone [1]. To mine such large amounts of textual information requires techniques that can handle this data efficiently. Clustering of data is the most fundamental technique that is used to group similar items in a cluster (or group). Text clustering finds various applications [3] such as web search results clustering, automatic document organization (and browsing), and social news clustering [47, 49]. It can also be used as an intermediate step for applications such as multi-document summarization [38, 44], real-time text summarization [23], sentiment analysis, topic extraction and labelling of documents.

The basic approach to performing document clustering is to use the Bag of Words (BOW) [25] model. In this approach, a vocabulary of unique words from the complete collection of documents (corpus) is formed. Then each document is numerically represented in terms of this vocabulary where each vocabulary term is assigned a score in a particular document. The scoring scheme can be straightforwardly the frequency of each word or schemes like TF-IDF [37] where term frequency (TF) is multiplied with inverse document frequency (IDF). As a result of this, a term-document matrix is formed on which a partitioning-based, hierarchical or any other kind of traditional clustering methods [40] is applied.

However, there are two major limitations of these conventional approaches. First, because the scoring schemes are just based on statistical measures like frequency, the actual semantics (meaning) of the words are not taken into account due to which problems of polysemy (the same word with different meanings in different contexts) and synonymy (different words having the same meaning) are not handled. Second, a well-known problem of high dimensionality known as the curse of dimensionality exists in this approach. This problem is more exaggerated when the number of documents in the corpus is very large, say in thousands or more. Hence, the number of dimensions can reach anywhere from tens of thousands to a few million, for a dataset containing some thousands of documents (depending upon the vocabulary size). In addition, the matrix representation of such datasets becomes very sparse (containing a large number of zeros). Traditional clustering techniques such as partitioning-based, hierarchical, and density-based cannot perform well under these circumstances because the dimensionality that they can handle is limited to a few hundred. In some cases, they even fail to perform clustering. To solve the problems of polysemy and synonymy, ontologies such as WordNet have been used in various research works [18, 29, 42, 46]. However, these approaches are highly dependent on word coverage and the design of WordNet [31]. Additionally, these approaches are mainly useful for only a few languages.

In this paper, it has been attempted to solve the aforementioned challenges using a relatively new concept called word embeddings. A word embedding is nothing but just a vector that represents a word in a document. It is a distributed (dense) representation of words using real numbers instead of the discrete representation using 0’s and 1’s. The dimensionality of this vector generally lies from hundreds to thousands. The initial algorithm to generate these embeddings is known as the Word2Vec algorithm developed by Tomas Mikolov in 2013 at Google [30]. The idea behind Word2Vec is to optimize an objective function such that the probability of a central word in a context window of a fixed size *m* is maximized. This is done by training a neural network architecture for a large corpus of text. The output of the network architecture is a numerical vector (or embedding) corresponding to a word. Other algorithms for word embeddings are Glove developed by Stanford university [36] and FastText by Facebook [8]. These all are open source projects and thus can be freely downloaded.^1^$$^{,}$$^2^ Several recent studies present a good survey on word embeddings [5, 7, 9, 45].

However, the aforementioned algorithms provide a fixed representation (embedding) for a word, which means the embeddings for a word are not context-based. For example, the word “bank” has different meanings based on the context in which it is used. Thus based on the context that the user provides as input, the model does not generate two different embeddings. In this research paper, a recently proposed model for generating contextual embeddings is used, known as Bidirectional Encoder Representations using Transformers (BERT) [12]. BERT is a complex neural network architecture that is trained on a large corpus of books and English Wikipedia. In this research paper a novel document clustering technique is proposed based on word embeddings derived using BERT. The proposed technique is called as WEClustering. WEClustering has the following features.

i. It deals with the problem of very high dimensionality that arises when dealing with text datasets with a large vocabulary.
ii. It is a context-sensitive semantic approach based on word embeddings derived from the BERT model.
iii. It yields high accuracy compared to several other widely used clustering approaches.

The proposed text clustering technique named WEClustering gives a unique way of leveraging the word embeddings to perform text clustering. This technique tackles one of the biggest problems of Text mining which is called the curse of dimensionality in its own way so as give more efficient clustering of textual data, especially suitable to the case of big textual datasets. To the best of our knowledge, it is one of the few clustering methods that exist in the literature so far that leverages BERT in a unique way. The proposed technique is validated using several textual datasets using different performance metrics. Further, to demonstrate its effectiveness, the results of the proposed technique are compared with the results of several widely used and state-of-the-art clustering techniques. The complete paper has been divided into five sections. “Related work and background” contains the work which directly relates to and serves as a background for the proposed clustering approach. “WEClustering: the proposed clustering technique” presents the architecture and detailed description of the proposed approach. “Implementation details, testing and result analysis” presents all the experiments conducted to validate the proposed approach. “Conclusions and future scope” concludes the whole work.

## Related work and background

In this section, significant details of the work that relates closely to the proposed clustering technique WEClustering are presented. This includes the architecture and workflow of BERT, the K-means algorithm, and its minibatch version for handling large datasets and agglomerative clustering algorithm. These techniques are the important components of WEClustering.

### Bidirectional Encoders representations using Transformers (BERT)

**Fig. 1:**
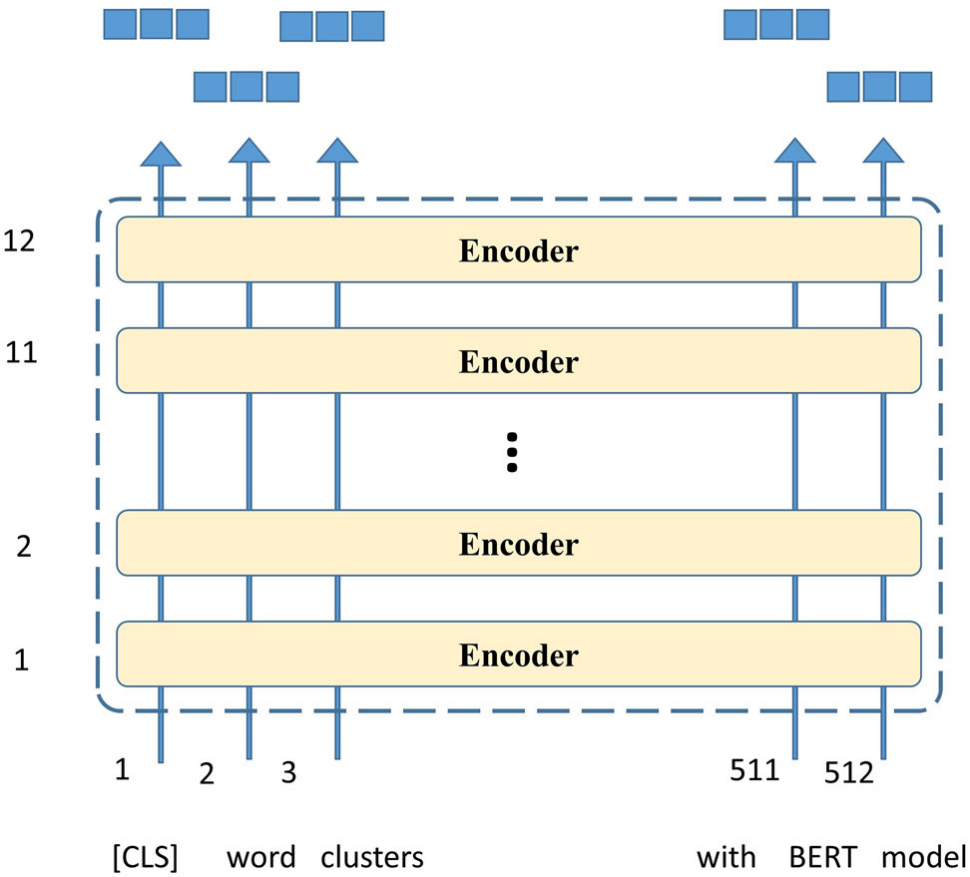
Architecture of BERT [4]


Representation of words and sentences in a way that can truly capture their meaning according to the context in which they fall is a rapidly evolving area of research in the field of Natural Language Processing (NLP). An important recent milestone in this direction was reached in late 2018 with the introduction of BERT. BERT is a deep learning model that made new records in dealing with language-based tasks such as sentence/sentiment classification, question answering system, and Named Entity Recognition (NER). Soon after the paper release [12], various versions of BERT have been open-sourced.^3^ These versions are already pre-trained on huge datasets of books and Wikipedia. Hence, one can use these models as it is or also can fine-tune them for different supervised task mentioned before, to generate context-based embeddings. A high-level architecture of the BERT model is shown in Fig. 1 [4]. It is a stack of transformer (encoder) layers. Two architectures BERT_base_ and BERT_large_ with 12 and 24 encoder layers respectively have been proposed in the original paper. The model takes as input a sequence of words, the first of which is a special token represented as “[CLS]”. The minimum length of the input sequence can be 1 and the maximum length is 512. Each encoder layer of BERT outputs a vector which is passed as input to the layer above it. For each word of the input sequence, the BERT_base_ and BERT_large_ models give a vector of length 768 and 1024 respectively as its final output. These vectors encode in them, the semantics of words and the relationships among words. These vectors can be used for different supervised downstream tasks such as question answering systems and sentiment analysis. This is generally done by adding a neural network layer plus a softmax function at the end of the model. The original paper reports outstanding results for these kinds of tasks in comparison to other state-of-the-art models. Since its release, BERT has been used in several text classification tasks [2, 11, 21, 32, 34]. For text clustering, only a few research papers exist in literature [22, 35, 43]. However, these techniques are not able to reduce the high dimensionality significantly. For example, in [22], a fixed number of dimensions (768) is used to represent all the documents in a dataset of any size whereas the proposed technique in this paper uses dimensionalities less than a hundred for all the datasets taken in the experiments. Moreover, this dimensionality is decided according to the dataset in hand using a suitable method as explained later. Second, the proposed WEClustering exploits the semantic relationships between words in its third phase (clustering of embeddings) to combine the words with similar meanings. This removes the problems of synonymy and polysemy which leads to increased accuracy. The focus of the proposed clustering technique WEClustering in this paper is to simultaneously deal with challenges such as synonymy, polysemy, high dimensionality, and provide high accuracy. This idea is much unique in comparison to the aforementioned clustering techniques using BERT.

### Minibatch K-means clustering

K-means is a widely used partitional clustering algorithm in which the sum of squares of distances between the center of a cluster and other data points of the cluster is minimized to obtain an optimal data partition of a given dataset [24]. Minibatch K-means [41] is a variant of the standard K-means algorithm, in which mini batches are used to optimize the same objective function. A minibatch is a subset of a complete dataset drawn randomly. For one training iteration, this minibatch is used instead of the complete dataset. As a result of this, the computation time for convergence of the algorithm to an optimal value is greatly reduced while the difference between the quality of clusters is reported to be only a little less than the original algorithm. Algorithm 1 shows the different steps involved in Minibatch K-means.

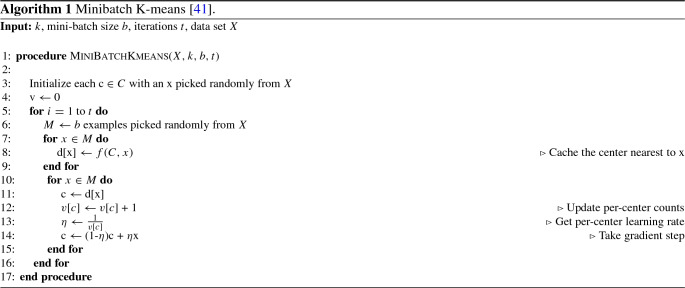


### Agglomerative clustering

Agglomerative clustering is a kind of bottom-up hierarchical approach to clustering. Initially, each data point is regarded as a cluster on its own, then two different clusters are merged that lie at the shortest distance among all the pairs of clusters. This merging is performed until either a single cluster remains or termination criteria is satisfied. An abstract form of agglomerative clustering is shown in the following steps:

i. Let each data point be a cluster on its own.
ii. Compute the proximity matrix of individual points.
iii. Merge the two closest clusters and then update the matrix.
iv. Repeat step iii. until a single cluster remains.

The output of this algorithm is a tree-like structure called a dendogram. This structure can be cut down at different levels to give different corresponding clusters. How inter-cluster distance is defined is known as linkage criteria. Four widely used linkage criteria found in the literature [33] are given as follows:

i. Complete linkage: The distance between the farthest pair of data points in two clusters is used for measuring inter-cluster similarity.
ii. Average linkage: The distance between the group averages of all data points in a cluster is used as a measure of inter-cluster similarity.
iii. Single linkage: The distance between the closest pairs of data points in clusters is used to measure inter-cluster similarity.
iv. Ward linkage: In this method, a pair of clusters is chosen for merging that minimizes the sum of intra-cluster variances for all the clusters [16]. In this research work, this variant is used as it produces more compact clusters.

## WEClustering: the proposed clustering technique

**Fig. 2:**
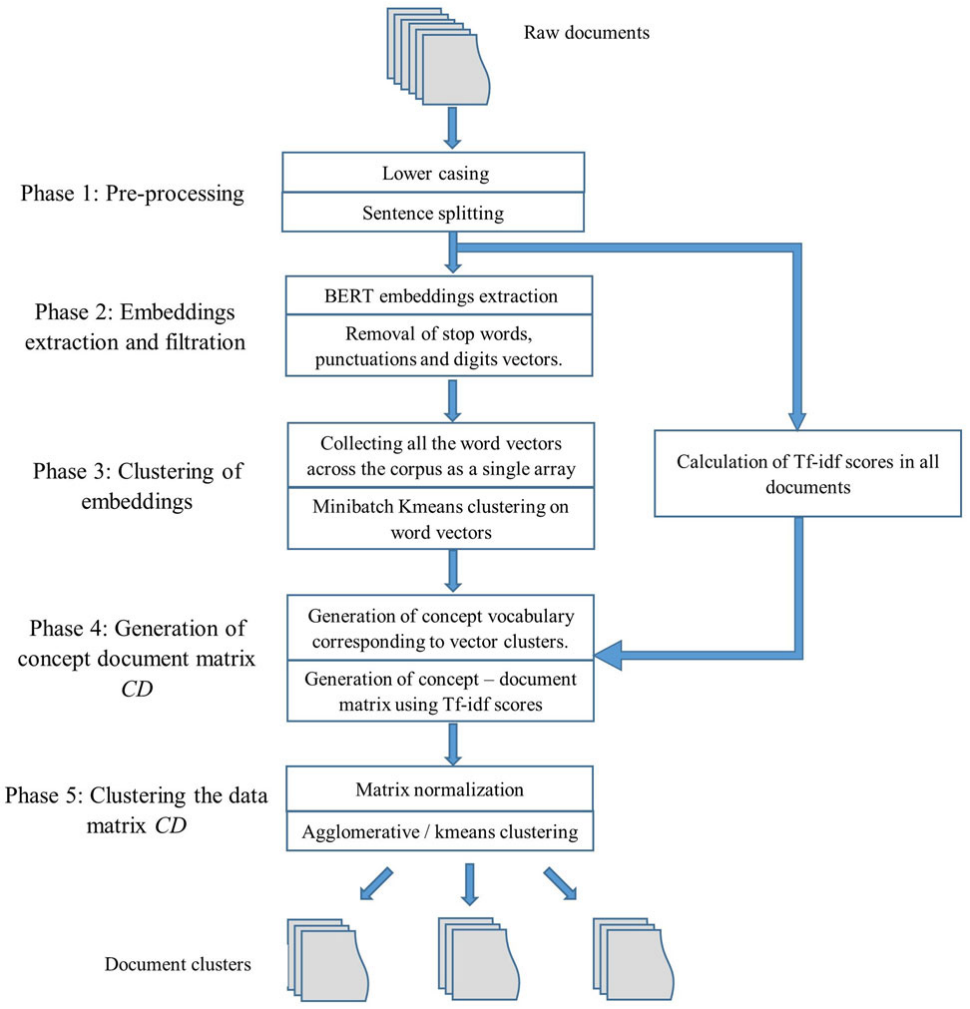
Flowchart of the proposed technique WEClustering

In this section, a detailed description of the proposed clustering technique called WEClustering is given. WEClustering combines the semantic advantages of the contextual word embeddings derived from the BERT model with statistical scoring mechanisms. The technique is divided into five different phases as shown in Fig. 2. The motivation behind the design of WEClustering as such is that TF-IDF scoring can capture the statistical importance of each word with respect to a document as well as the whole corpus, while BERT embeddings can capture context-based semantics of each word very well. Hence, WEClustering provides a unique way of combining the statistical and semantic features of the text. Each phase is described as follows.

i. Pre-processing: In this first phase, all the documents are prepared in a format suitable for processing with BERT. Although BERT is capable of producing case-sensitive embeddings for words, however, all the documents are converted in lower case for simplicity. As BERT finds contextually dependent embeddings, it takes a complete sentence as its input. Hence in the next sub-step of this phase, all the documents are split into sentences.
ii. Embeddings extraction and filtration: In this phase, firstly, the pre-processed data is fed into the pre-trained (weight parameters are fixed) BERT model. As a result of this, each word of all the documents is converted into a vector (embedding) of size 1024. Second, embeddings that are not so semantically important and hence do not play role in discriminating the documents are removed. These include embeddings corresponding to digits, punctuations, and stop words.
  The steps i. and ii. are shown in the form of Algorithm 2.

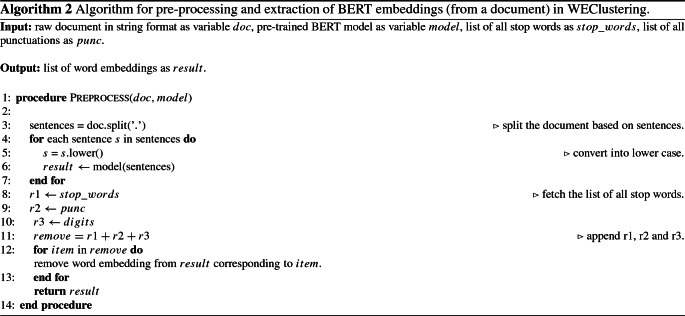

iii. Clustering of word embeddings: The conversion of all the words (string format) into a numerical vector format makes it very easy and accurate to measure similarity (or dissimilarity) between words. This kind of semantic comparison between words was not much accurate before the introduction of models like BERT. Hence, in this research, the difference in semantics of words based on embeddings is leveraged to form document clusters. In this phase, all the word vectors achieved out of phase (ii) are arranged in the form of a matrix of dimension (no. of words $\times$ 1024). Then, Minibatch K-means clustering is applied to this matrix. As a result, clusters of words, now onwards called a “concept” are formed. These clusters (concepts) represent a unique theme contained in some documents. The idea is to use these concepts as the new vocabulary (or features) instead of individual words as a vocabulary to represent any document. The total size of the vocabulary will be equal to the number of clusters denoted as $k_{\text {voc}}$. This value is chosen using the Elbow method [17]. In the Elbow method, firstly, a performance metric like the Silhouette coefficient is plotted against a range of values of ‘k’ (number of clusters). Then the first most significant turning point is taken to be the number of clusters. As a result of the clustering of embeddings, the size of vocabulary gets reduced drastically from tens of thousands to less than a hundred. This step is shown in the form of Algorithm 3. The reason behind choosing this algorithm is that it drastically reduces the computational time as compared to the standard K-means algorithm. As the name suggests, it uses mini batches instead of the complete dataset for each iteration of training.

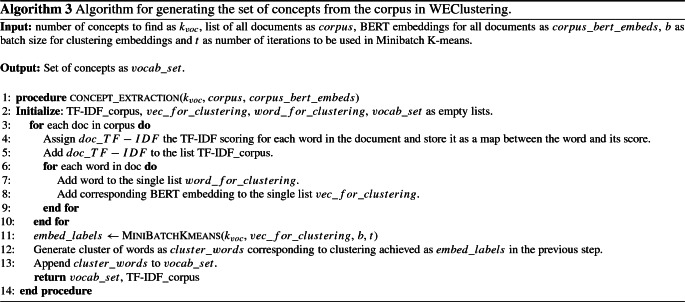

iv. Generation of concept-document matrix *CD*: After generating concepts in phase (iii), each document now is represented in terms of all the concepts. As a result, all the documents of a corpus are collectively represented in the form of a matrix which is hereafter called a Concept-Document (CD) matrix. Each concept is given a score in each document to represent its degree of relation to that document. The scoring mechanism for an *i*th document $d_i$ for *j*th concept $c_j$ is represented by ${\text {CD}}_{ij}$ which is defined as follows.
  1 $$\begin{aligned} {\text {CD}}_{ij} = \sum _{k} {\text {TF-IDF}}(w_{jk}), \end{aligned}$$
  where
  2 $$\begin{aligned} {\text {TF-IDF}}\left( w_{jk}\right) = {\text {freq}}\left( w_{jk}\right) \times \left( \log \left( \frac{ |D| + 1}{{\text {doc}}\_{\text {count}}\left( w_{jk}\right) +1}\right) + 1\right) . \end{aligned}$$
  Here, TF-IDF values of all *k* words contained in the concept $c_j$ corresponding to the document $d_i$ are added together. |*D*| is the total number of documents in the corpus *D*, ${\text {freq}}(w_{jk})$ is the frequency of word $w_{jk}$ in document $d_i$ and ${\text {doc}}\_{\text {count}}(w_{jk})$ is the total number of documents that contain the word $w_{jk}$. The size of the matrix CD comes out to be (no. of documents x vocabulary size).
v. Clustering the data matrix CD: In this final phase, document clustering is performed by applying a traditional clustering technique such as agglomerative clustering or K-means on the CD matrix. Because the number of features that are used to represent a document is drastically reduced, a traditional algorithm like hierarchical agglomerative clustering or K-means performs nicely on the input matrix. As a result of this phase, well-separated clusters of documents are achieved.
  The algorithm for steps iv. and v. is presented in Algorithm 4.

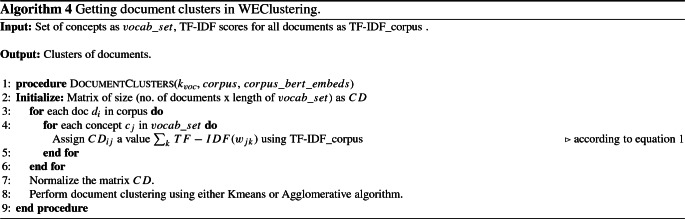


## Implementation details, testing and result analysis

To validate the effectiveness of the proposed WEClustering technique, it is implemented on several real-world textual datasets. This section gives necessary experimental details of its implementation. Second, a comparison of the results with other widely used and state-of-the-art text clustering techniques is presented to demonstrate its efficiency over other techniques. Clustering results are presented with the help of suitable performance metrics such as silhouette coefficient, ARI, and Purity.

### Datasets used

A total of seven benchmark real-world datasets of different sizes and domains are used for assessing all the techniques. Relevant details of these datasets are given below and are also summarized in the form of Table 1.

i. Articles-253
  This corpus^4^ is a collection of five different categories of research articles. Each document consists of title, abstract, and references. The categories of this dataset correspond to the publication houses from which they are obtained. These are Transactions on Mobile Computing, American Political Science Review, Monthly Weather Review, British Food journal, and DNA research. The number 253 in the title depicts the total number of articles in this dataset.
ii. Scopus
  This dataset is a part of a complete dataset and contains 500 articles. These articles are equally divided into five categories namely ‘concrete’, ‘hyperactivity’, ‘investment’, ‘photosynthesis’, and ‘tectonicplates’. As per its name, it is obtained from the Scopus database and each document consists of a title and an abstract (see footnote 4 to download the complete dataset).
iii. 20NG
  This dataset is a subset obtained out of a widely used 20 newsgroups dataset^5^ which consists of news articles of 20 different categories. The subset of categories included for this dataset are ‘alt.atheism’, ‘talk.religion.misc’, ‘comp.graphics’, and ‘sci.space’. The total number of documents in this dataset is 700.
iv. Classic4
  This collection is made up of research articles of different domains which are aerodynamics, medical, computing algorithms, and information retrieval. However, in implementation, only the first three categories are included because in the fourth category documents were very short. The total number of documents in this corpus is 800 (see footnote 4 to download).
v. Scopus-long
  This is a collection of 2800 research articles from the Scopus database containing the titles and abstracts. All the documents are equally divided into 7 categories each containing 400 articles. The categories are ‘investment’, ‘neural network’, ‘hyperactivity’, ‘concrete’, ‘proton’, ‘photosynthesis’ and ‘tectonic plates’ (see footnote 4).
vi. Classic4-long
  This is a large version of the Classic4 dataset consisting of 3891 documents.
vii. 20NG-long
  This is a large part obtained from 20 newsgroups dataset (footnote 5). The categories included in this dataset are ‘alt.atheism’, ‘talk.religion.misc’, ‘comp.graphics’, ‘sci.space’, ‘rec.motorcycles’, ‘rec.sport.hockey’, ‘sci.med’, ‘sci.electronics’, and ‘talk.politics.misc’. Total number of documents in this corpus are 8131 divided into aforementioned 9 categories.

**Table 1:** Properties of datasets used in experiments

S. no.	Dataset	Total categories	Total documents
1.	Articles-253	5	253
2.	Scopus	5	500
3.	20NG	4	700
4.	Classic4	4	800
5.	Scopus-long	7	2800
6.	Classic4-long	4	3891
7.	20NG-long	9	8131

### Comparison schemes

For measuring the efficiency of the proposed technique over existing techniques, a comparison with the following clustering techniques based on the Bag of words model representation of documents is performed.

i K-means: It is a popular partitioning-based [19] clustering algorithm. Originally, it was proposed in 1967 [14] but because of its simplicity and less computational cost, it is widely used still.
ii Agglomerative clustering: This is a hierarchical type of clustering algorithm [48] in which data points are combined to gradually form clusters to give a tree-like structure known as dendogram [40]. This dendogram is cut at a specified level to give the required clusters.
iii Hierarchical Density-based spatial clustering of applications (HDBSCAN): This algorithm [27, 28] is a robust variant of the density-based clustering algorithm DBSCAN [13]. It is a quite recent clustering technique that showed better performance than several other algorithms.
iv Genie: It is quite a recent hierarchical clustering algorithm [15] which performs clustering based on Gini index [10] (a popular statistical measure used for measuring dispersion in a given list of frequency values). Genie makes sure that the value of the Gini index should not exceed a given threshold. If it exceeds then the smallest cluster is merged with its nearest neighbor.
v Disambiguated core semantics (DCS): This approach [46] used lexical chains (groups of semantically related words derived using WordNet) to find the most important concepts in a document. Subsequently, K-means is applied to this reduced set of concepts to get the document clusters.
vi Stamantic Clustering (STC): This technique [29] performs document clustering by combining statistical and semantic features using TF-IDF as a scoring scheme and exploiting semantic relations using WordNet.

### Parameter settings

**Fig. 3:**
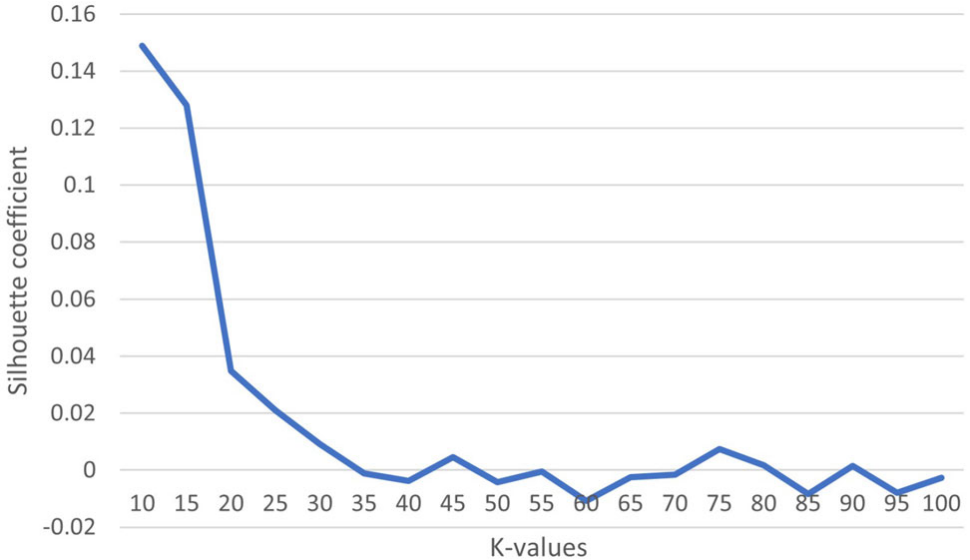
Description of Elbow method on Articles-253 dataset to find $k_{\text {voc}}$

Different parameter settings used in different phases of the proposed clustering technique (WEClustering) are explained below.

i. Embeddings extraction: Two different BERT models are available to use:
  - BERT_small_, that generates embedding vectors of size 768. Further, this can be case sensitive or case insensitive.
  - BERT_large_, that generates embedding vectors of size 1024. This is available as the only case-sensitive model.^6^
    In our approach, BERT_large_ is used in the embeddings extraction and filtration phase because word semantics are captured better in the higher dimensional vectors.
ii. Clustering of embeddings: In this (third) phase of the technique, the Minibatch K-means algorithm is used to perform clustering of embeddings. Important parameters of this algorithm are the number of clusters of words $k_{\text {voc}}$ and the batch size *b*. Table 2 lists the values of all these parameters used for all seven datasets. For the first six datasets, a value of 25 or 35 has been determined using the Elbow method as already described in “WEClustering: the proposed clustering technique”. As an example, the Elbow method for the Articles-253 dataset has been shown with the help of Fig. 3 in which silhouette coefficient is plotted against the range [10, 100] of *k* values. The most significant turning point (elbow) is detected for the value of 35. A similar approach is used for other datasets as well. The batch-size parameter *b* does not much affect the clustering accuracy as given in the original paper [41] and hence does not require any formal optimization in our work. The values of *b* are so chosen that they are close to the values used in the original paper [41] and the execution time is as less as possible.
iii. Document clustering: In the last phase of the proposed clustering technique that is document clustering, a simple clustering algorithm such as Agglomerative clustering or partitioning algorithm such as K-means is used. In Agglomerative clustering, the important parameter is the ‘linkage’ as defined in “WEClustering: the proposed clustering technique”. In this paper, ward linkage is used to complete the process of document clustering. While using K-means clustering, the parameter *c* that is the number of clusters is the number of classes/categories contained in the dataset. Also, as K-means is sensitive to the initialization of the centroids, the method of K-means++[6] is used for initialization. Additionally, the number of times it is run is 10 and the best result is reported.

K-means, Agglomerative, Genie, DCS, and STC require the number of clusters as the only parameter. In our experiments, the number of clusters is equal to the number of available classes for each dataset. HDBSCAN returns a good clustering straight away with little or no parameter tuning. The primary parameter, i.e. minimum cluster size is intuitive and easy to select. It is set to the smallest size grouping that one wishes to consider a cluster. However, different values of this parameter may produce a different number of clusters than the actual number of classes. Hence, we took that minimum value which produced the actual (i.e. the number of classes) number of clusters.

**Table 2:** Values of parameters $k_{\text {voc}}$ and batch size *b* used for datasets in phase iii

S. no.	Dataset	$k_{\text {voc}}$	*b*
1.	Articles-253	35	5000
2.	Scopus	35	5000
3.	20NG	25	5000
4.	Classic4	35	5000
5.	Scopus-long	35	5000
6.	Classic4-long	35	5000
7.	20NG-long	75	25,000

### Performance metrics

Several metrics are defined in the literature to assess the quality of clustering. Based on the availability of ground truth labels, they can be classified as external (when true labels are available) and internal (when true labels are not known). For assessment of the results produced in this research study, the following metrics are used.

i. Silhouette coefficient: This is a widely used metric when true labels are not available which is the actual case in a clustering task. It is a measure of how dense and well separated the clusters are. Its mathematical formulation is given as:
  3 $$\begin{aligned} s=(b-a)/\max (b-a), \end{aligned}$$
  Where *a* is the average distance between a sample and all other points in the cluster and *b* is the average distance between a sample and all other points of the next nearest cluster. Its range lies between − 1 and + 1 (both inclusive). A higher value indicates dense and well-separated clusters.
ii. Adjusted Rand Index (ARI): ARI is a widely used metric for assessing cluster quality in the case of availability of true labels [39]. It can be used to measure two different clustering assignments that ignore different permutations of the same clustering. Two similar clusterings achieve a score near + 1.0 and completely different clusterings achieve a score approaching − 1.0.
iii. Purity: This measure is also an external measure that calculates the quality of clustering by first assigning all the data points in a cluster to the class for which the maximum number of data points are present in this cluster. This is done and summed over all the clusters and then normalized by the total number of data points [26].

### Analysis of results

**Fig. 4:**
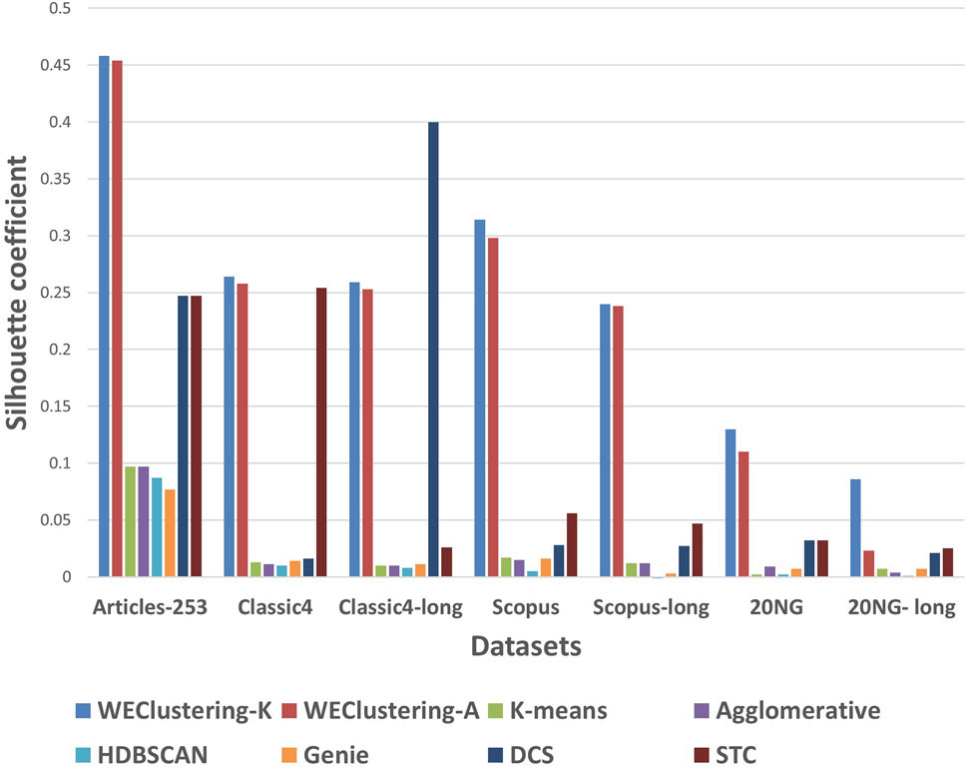
Column chart indicating the values of Silhouette coefficient for clustering techniques

**Fig. 5:**
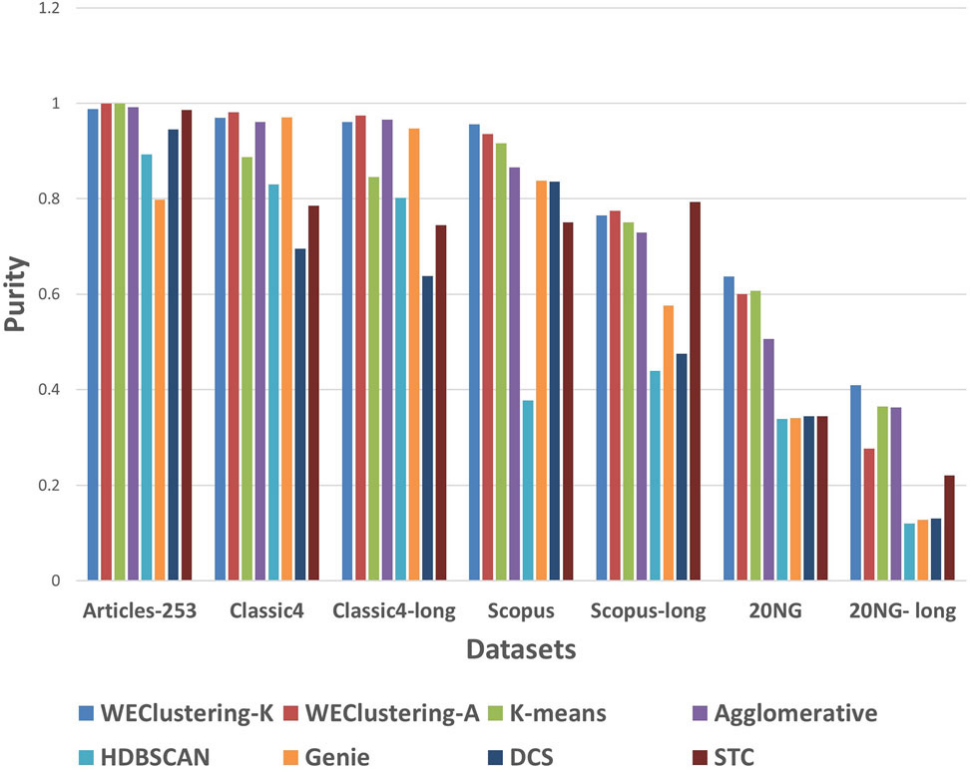
Column chart indicating the values of purity for clustering techniques

**Fig. 6:**
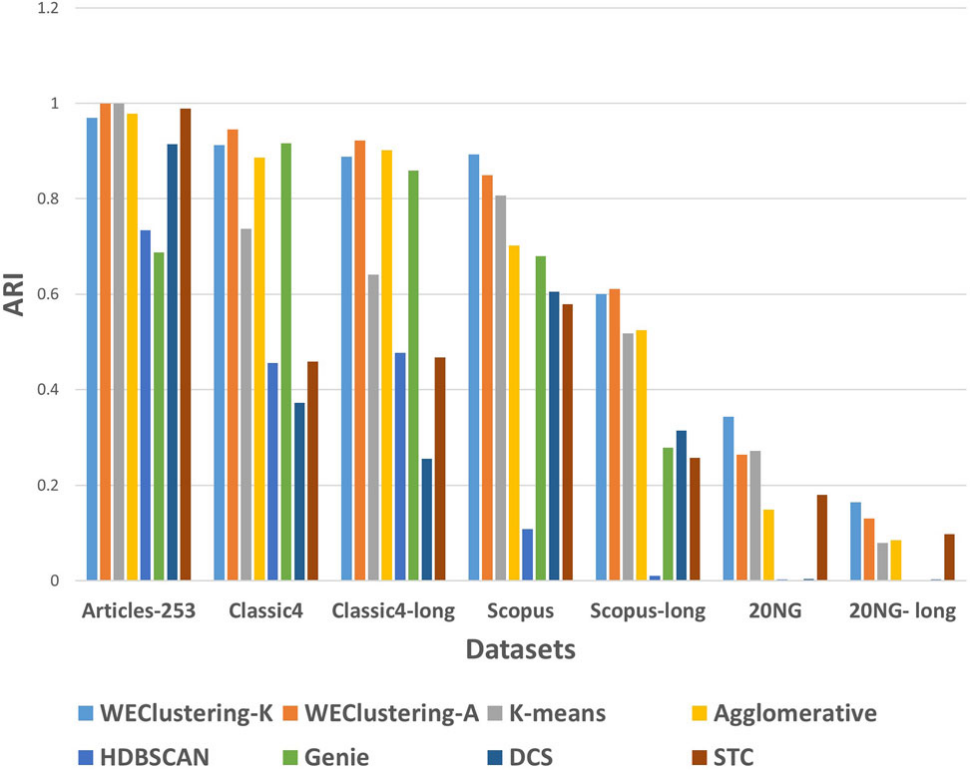
Column chart indicating the values of ARI for clustering techniques

**Fig. 7:**
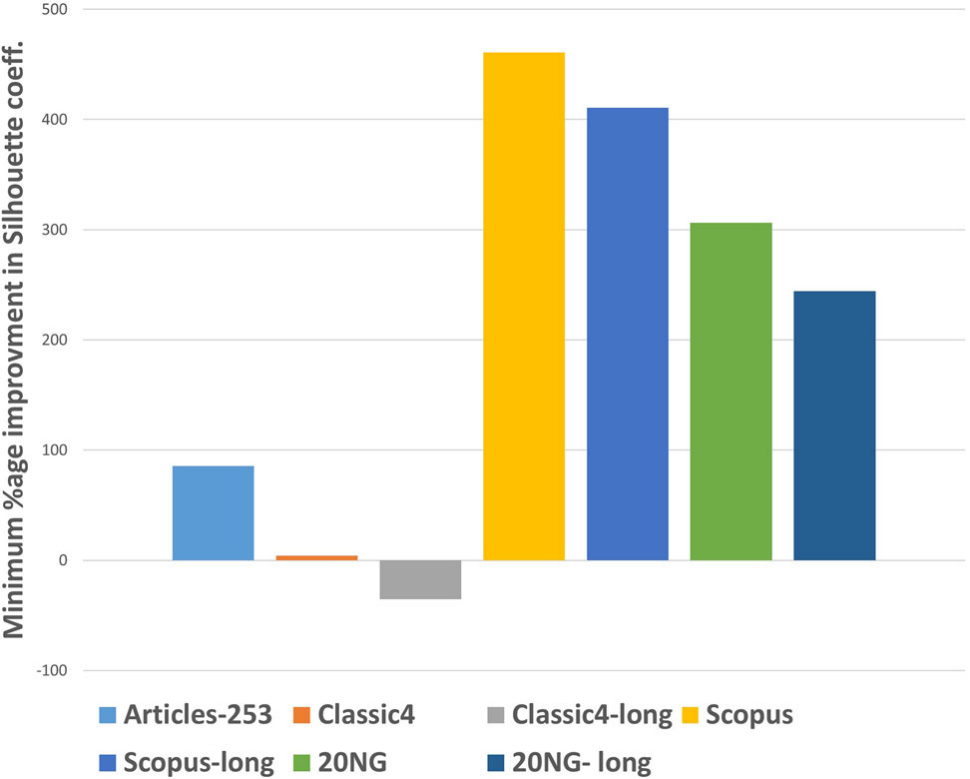
Column chart indicating the improvement of performance using the proposed clustering technique w.r.t Silhouette coefficient

**Fig. 8:**
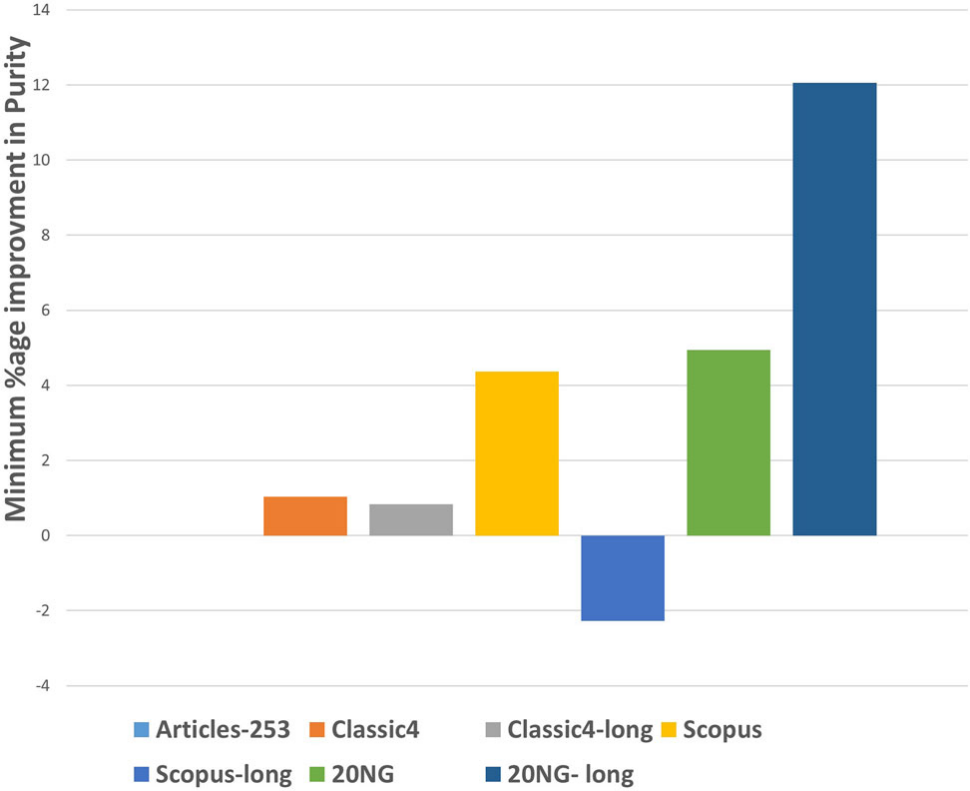
Column chart indicating the improvement of performance using the proposed clustering technique w.r.t purity

**Fig. 9:**
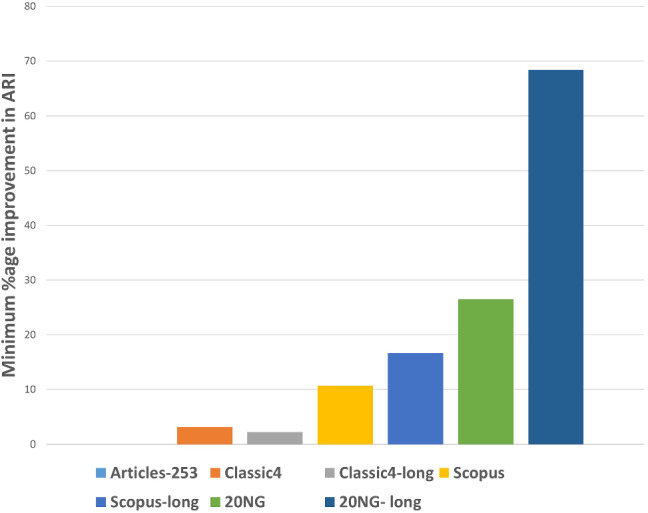
Column chart indicating the improvement of performance using the proposed clustering technique w.r.t ARI

After executing the proposed clustering technique WEClustering and the other aforementioned techniques, many important results are achieved. In this subsection, the results of WEClustering and its comparative analysis with other techniques are presented in detail with the help of suitable graphics and tables. In tables and text, WEClustering^K^ and WEClustering^A^ specifically denote the use of K-means and agglomerative clustering respectively in the last phase of WEClustering. Analysis for each performance metric is as follows.

i. Silhouette coefficient: Table 3 shows the performance of all the techniques based on the Silhouette coefficient. This metric is the most important of all the three performance metrics because, in a real scenario of clustering, true labels are not available to us. As already mentioned, the Silhouette coefficient determines the quality of clusters without requiring external labels. For all the seven datasets, the value of this metric goes much higher for the proposed clustering technique except for one dataset which is classic4-long. This is probably due to the reason that DCS also can reduce the dimensionality as it tries to find the core concepts5. Best values are indicated in bold. To get some more insights a column chart corresponding to the Table 3 is plotted in the form of Fig. 4. It makes it very clear that WEClustering with either K-means or agglomerative clustering in its final phase, both outperforms all other clustering techniques. Additional minute details of performances can be seen with the help of Table 6 and Fig. 7. Table 6 highlights the minimum %age improvement made by the proposed clustering technique over other techniques for all the datasets and performance measures. Figure 7 shows a column chart corresponding to this improvement in terms of the silhouette coefficient. Minimum %age improvement is defined in this paper as
  4 $$\begin{aligned}&\min \_\%{\text {age}}\_{\text {improvement}} \nonumber \\&\qquad \qquad = 100 \times \frac{\text {Score}_{\text {proposed}} - {\text {MaxScore}}_{\text {others}}}{\text {MaxScore}_{\text {others}}}, \end{aligned}$$
  where $\text {Score}_{\text {proposed}}$ is the score achieved by the proposed technique, ${\text {MaxScore}}_{\text {others}}$ is the maximum score of all other techniques For example, the silhouette coefficient score achieved by WEClustering^K^ for dataset Articles-253 is 0.458 and the maximum score among all scores of all other four techniques is 0.097. Hence, according to Eq. 4, minimum %age improvement becomes 372.164. A very large %age improvement can be seen for the Silhouette coefficient in all the datasets especially for datasets containing a larger number of documents. This can be attributed to the fact that by its definition silhouette coefficient measures how well separated and dense the resulting clusters are formed. As the proposed technique can reduce the dimensionality of datasets from tens of thousands to less than 100, the resulting clusters formed in the last phase are quite well separated and dense.
ii. Purity: For the case when external labels are available, purity can be used to measure the clustering results. Table 4 highlights the purity values achieved by all the clusterings for all the datasets. Again, it is very clear that WEClustering outperforms all other techniques except for one dataset. A visualization chart corresponding to these values is provided in Fig. 5. In column 4 of Table 6, values of %age improvement gained by WEClustering over others are provided. This improvement is calculated in the same way as for silhouette coefficient, i.e. using Eq. 4. Figure 8 shows a column chart corresponding to these improvement values. It can be inferred from the table that for each of the datasets, there is a significant performance improvement. For the Articles-253 dataset, WEClustering^A^ and K-means achieved the maximum purity score, i.e. 1.0, therefore improvement comes out to be zero. It should also be noticed from the figure that as the size of the dataset grows, more improvement performance is taking place. This trend proves the efficiency of the proposed technique for large datasets.
iii. ARI: To more verify the results achieved as measured by the above two performance metrics, a third metric called ARI is also used. As aforementioned, like purity, it measures performance with respect to available ground truth labels. Individual values of ARI for clustering achieved by different clustering algorithms for all datasets is shown in Table 5. It can be seen that for Articles-253, WEClustering^A^ and K-means both achieved the maximum ARI score of 1.0. It is probably because it is a small dataset containing only 253 articles, so it is easy to find clusters in it. For all other datasets, WEClustering achieves greater ARI values. The best values are shown in bold. Figure 6 depicts the same trend with the help of a column chart. Additionally, minimum %age performance improvement in terms of ARI is shown with the help of Fig. 9. Again, it can be seen that there is a significant performance improvement made by the proposed clustering technique WEClustering. Also, as one goes from smaller to larger datasets, performance improvement increases.
  The reason behind these results can be attributed to the fact that word embeddings derived from the BERT model capture the semantics of the word and its context better. Other clustering techniques that are just based on a scoring mechanism like TF-IDF cannot capture the meaning of a word with respect to its context. The proposed technique combines the advantages of statistical scoring mechanisms like TF-IDF as well as the semantics of the word. Additionally, the clustering of word embeddings using Minibatch K-means combines the words with similar contexts into a single group or a cluster. Hence, it reduces the dimensionality of the problem drastically i.e. from tens of thousands to less than a hundred. This enables the formation of more accurate clusters. Additionally, it is worth mentioning that the 20NG-long dataset shows the lowest performance for all the clustering techniques in comparison to other datasets. The reason for the worst performance on this dataset can be attributed to the fact the categories under which the dataset is divided significantly overlap with each other. For example, “sci.med”, “sci.space”, “sci.electronics” are three different sub-categories falling under the single category “science”. Similar is the case with “rec.motorcycles” and “rec.sport.hockey”. Moreover, the individual documents are not much longer to provide sufficient information to discriminate among them.
  The difference between WEClustering^K^ and WEClustering^A^ can be due to the reason that in the last phase of WEClustering, K-means and Agglomerative clustering are applied respectively. K-means and Agglomerative clustering belong to two different categories i.e. Partitioning-based and Hierarchical-based respectively, hence little difference has appeared in a performance of WEClustering^K^ and WEClustering^A^.
iv. Execution time: The time taken in WEClustering to form clusters using its low dimensional concept-document matrix representation for each dataset is shown in Table 7. Similarly, the time taken by other techniques to form clusters using the high-dimensional term-document matrix representation for each dataset is also shown. WEClustering exhibits the lowest execution time for each dataset. The time to form clusters directly depends on the number of dimensions used, hence the values listed in Table 7 for WEClustering are much lower than any other clustering technique.

**Table 3:** Silhouette coefficient values of WEClustering and other techniques on different datasets

S. no.	Datasets	WEClustering^K^	WEClustering^A^	K-means	Agglomerative	HDBSCAN	Genie	DCS	STC
1.	Articles-253	**0.458**	0.454	0.097	0.097	0.087	0.077	0.247	0.247
2.	Classic4	**0.264**	0.258	0.013	0.011	0.010	0.014	0.016	0.254
3.	Classic4-long	0.259	0.253	0.010	0.010	0.008	0.011	**0.400**	0.026
4.	Scopus	**0.314**	0.298	0.017	0.015	0.005	0.016	0.028	0.056
5.	Scopus-long	**0.240**	0.238	0.012	0.012	− 0.016	0.003	0.027	0.047
6.	20NG	**0.130**	0.110	0.002	0.009	0.002	0.007	0.032	0.032
7.	20NG-long	**0.086**	0.023	0.007	0.004	0.001	0.007	0.021	0.025

**Table 4:** Purity values of WEClustering and other techniques on different datasets

S. no.	Datasets	WEClustering^K^	WEClustering^A^	K-means	Agglomerative	HDBSCAN	Genie	DCS	STC
1.	Articles-253	0.988	**1.0**	**1.0**	0.992	0.893	0.798	0.945	0.986
2.	Classic4	0.970	**0.981**	0.887	0.961	0.830	0.971	0.695	0.785
3.	Classic4-long	0.961	**0.974**	0.845	0.966	0.802	0.947	0.638	0.745
4.	Scopus	**0.956**	00.936	0.916	0.866	0.378	0.838	0.836	0.750
5.	Scopus-long	0.765	0.775	0.750	0.729	0.439	0.576	0.475	**0.793**
6.	20NG	**0.637**	0.600	0.607	0.506	0.339	0.341	0.345	0.345
7.	20NG-long	**0.409**	0.277	0.365	0.363	0.120	0.128	0.131	0.221

**Table 5:** ARI values of WEClustering and other techniques on different datasets

S. no.	Datasets	WEClustering^K^	WEClustering^A^	K-means	Agglomerative	HDBSCAN	Genie	DCS	STC
1.	Articles-253	0.970	**1.0**	**1.0**	0.978	0.734	0.687	0.914	0.989
2.	Classic4	0.912	**0.945**	0.737	0.886	0.456	0.916	0.373	0.458
3.	Classic4-long	0.888	**0.922**	0.641	0.902	0.477	0.859	0.256	0.467
4.	Scopus	**0.893**	0.849	0.807	0.702	0.108	0.680	0.605	0.579
5.	Scopus-long	0.600	**0.611**	0.518	0.524	0.010	0.279	0.315	0.258
6.	20NG	**0.344**	0.264	0.272	0.149	0.003	0.001	0.004	0.180
7.	20NG-long	**0.165**	0.131	0.079	0.085	0.001	0.000	0.003	0.098

**Table 6:** Minimum %age improvement in performance using WEClustering

S. no.	Datasets	Min. %age improvement in Silhouette coefficient	Min. %age improvement in Purity	Min. %age improvement in ARI
1.	Articles-253	85.425	0	0
2.	Classic4	3.937	1.029	3.165
3.	Classic4-long	− 35.25	0.828	2.217
4.	Scopus	460.714	4.366	10.656
5.	Scopus-long	410.638	− 2.269	16.603
6.	20NG	306.25	4.942	26.470
7.	20NG-long	244	12.054	68.367

**Table 7:** Execution times (in seconds) of WEClustering and other techniques on different datasets

S. no.	Datasets	WEClus-tering^K^	WEClus-tering^A^	K-means	Agglomerative	HDBSCAN	Genie	DCS	STC
1.	Articles-253	0.082	0.009	10.692	0.275	2.387	0.353	0.842	1.168
2.	Classic4	0.107	0.022	13.018	1.142	5.358	1.462	2.966	32.55
3.	Classic4-long	0.266	0.638	73.029	70.011	270.604	71.791	32.537	3.332
4.	Scopus	0.152	0.010	11.053	0.464	2.606	0.558	2.280	2.516
5.	Scopus-long	0.060	0.258	2.120	1.140	203.293	44.779	1.151	48.04
6.	20NG	0.089	0.017	9.567	1.147	6.823	1.161	3.445	2.09
7.	20NG-long	2.274	5.223	129.195	1017.126	4886.316	803.929	40.256	35.38

## Conclusions and future scope

Document clustering is an important task in the field of text mining. Existing clustering techniques have some limitations when applied to textual datasets based on TF-IDF based term-document matrix. Recently proposed deep learning model, i.e. BERT can capture the semantics of a word very well especially with respect to the context in which it falls. In this paper, a novel and powerful document clustering technique named WEClustering is proposed that combines the advantages of statistical scoring mechanisms such as TF-IDF and state-of-the-art deep learning models such as BERT. WEClustering first extracts embeddings for all the words in a document using the BERT model and then combines them to form clusters of words with similar kinds of meanings and context. Based on these clusters of words which is called a concept in this paper, a concept document matrix is formed. Finally, this matrix is given as input to clustering algorithms such as K-means and agglomerative clustering which gives clusters of documents as the output. This process drastically reduces the problem of high dimensionality which is often encountered in the field of text mining or natural language processing. The technique is very well validated on seven different datasets containing documents ranging from a few hundred to several thousand in number. Based on different performance metrics, the proposed technique is compared with widely used and state of the art techniques such as K-means, agglomerative clustering, HDBSCAN, Genie, DCS, and STC. Results show that the WEClustering outperforms all the compared techniques. The minimum improvement reaches up to 90% in the case of larger datasets. As part of future work, it can be stated that the BERT model can be further fine-tuned to individual datasets to give better contextual word embeddings which can result in better clustering accuracy. Secondly, as the size of datasets keeps increasing day by day, the proposed technique can be tested on more large-sized datasets such as those containing millions of text documents.

## Footnotes

1 https://nlp.stanford.edu/projects/glove/.

2 https://fasttext.cc/.

3 https://github.com/google-research/bert.

4 Available at: https://vhasoares.github.io/downloads.html. Accessed: 2020-11-18.

5 http://qwone.com/~jason/20Newsgroups/.

6 pypi.org.
